# Supplementary material for: Immune Checkpoint Inhibitor Treatment and Ophthalmologist Consultations in Patients with Malignant Melanoma or Lung Cancer—A Nationwide Cohort Study
Source: Cancers (Basel). 2021 Dec 23;14(1):49. doi: 10.3390/cancers14010049 (PMC8750081; doi:10.3390/cancers14010049)
Supplement: Supplementary file 1 [file cancers-14-00049-s001.zip › cancers-1507133-supplementary.pdf]

Supplementary Table S1: Specification of immune checkpoint inhibitor treatment by procedure codes, definitions based on diagnoses by ICD-10 codes and ATC codes.

**Immune checkpoint inhibitor treatment**	Procedure code:<br>Ipilimumab: BIHJ19D,<br>Pembolizumab: BIHJ19J3,<br>Nivolumab: BOHJ19H2,<br>Atezolizumab: BOHJ19J2,<br>Durvalumab: BOHJ19H7
**Cancer types**	**ICD-10 codes**
Hodgkin's lymphoma	ICD-10: C81
Lung cancer	ICD-10: C34
Malignant melanoma	ICD-10: C43
Renal cell	ICD-10: C64, C65
Urinary tract cancer	ICD-10: C66, C67, C68
Head and neck	ICD-10: C00. C01, C02, C03, C04, C05, C06, C07, C08, C09, C10, C11, C12, C13, C14
Skin	ICD-10: C44
**Comorbidity**	**ICD-10 codes and other criteria**

Chronic kidney disease	ICD-10: E102, E112, E132, E142, I120<br>N02, N03, N04, N05, N06, N07, N08, N11, N12, N14, N18, N19, N26, N158, N159, N160, N162, N163, N164, N168, Q61, M321B
Diabetes mellitus	ICD-10: E10, E11, E12, E13, E14<br>ATC: A10 (6 months before AF diagnosis)
Hypertension	ICD-10: I10, I11, I12, I13, I14, I15<br><br>Usage of a combination of at least two of the seven different drug classes at the same time:<br><br>1. Non-loop diuretics<br>2. Loop diuretics<br>3. Antiadrenergic agents<br>4. Beta-blockers<br>5. Vasodilators<br>6. Calcium channel blockers<br>7. Renin-angiotensin system inhibitors
Morbus Bechterew	ICD-10: M459
Chronic tissue disease	ICD-10: M3
Inflammatory arthritis	ICD-10: M05-14
Juvenile arthritis	ICD-10: M08

Sarcoidosis	ICD-10: D86
Syphilis	ICD-10: A53
Multiple sclerosis	ICD-10: G35
Borrelia infection	ICD-10: A692
HIV	ICD-10: B20-24
**Ophthalmologist consultation**	Department code: 44 (variable name ‘afd’)

ATC: anatomical therapeutic chemical classification. ICD: international classification of diseases

Supplementary Table S2: Time from cancer diagnosis to initial ICI administration.

Cancer	Median	P25	P75
All	338	110	746
Lung cancer	1186	59	520
Malignant melanoma	505	205	975
Urinary tract cancer	377	207	930
Kidney	419	134	918
Head and neck	917	572	1368
Skin	1331	868	1808

Supplementary table 3: Absolute risks at 1 year after initial ICI-administration associated with type of immune checkpoint inhibitor

**Outcome**	**Subgroup** (N/included in analysis*)	**N** 365d	**Absolute risk [95% CI]** 1 year
Ocular inflammation	Ipilimumab (229/239)	6	2.6 [0.6-4.7]
Ophthalmologist consultation	Ipilimumab (196/239)	17	8.7 [4.8-12.7]
Ocular inflammation	Nivolumab (909/946)	NA	NA
Ophthalmologist consultation	Nivolumab (787/946)	24	5.7 [3.7-7.5]
Ocular inflammation	Ipilumumab + Nivolumab (116/122)	NA	NA
Ophthalmologist consultation	Ipilumumab + Nivolumab (89/122)	13	16.3 [8.1-24.5]
Ocular inflammation	Pembolizumab (787/804)	NA	NA
Ophthalmologist consultation	Pembolizumab (607/804)	24	5.0 [3.0-7.0]

*Subgroup analysis of type of cancer on patients naïve to the specific outcome previous to first ICI administration. E.g. in the analysis of ocular inflammation in patients with all cancers, 2119 out of 2190 patients did not have ocular inflammation previous to the first ICI-administration and were included in the analysis.
